# Retraction: EGFR Inhibition in Glioma Cells Modulates Rho Signaling to Inhibit Cell Motility and Invasion and Cooperates with Temozolomide to Reduce Cell Growth

**DOI:** 10.1371/journal.pone.0222210

**Published:** 2019-09-03

**Authors:** The PLOS ONE Editors

After publication of this article [1], the following concerns were raised:

- In Fig 4B, pRB panel (LN229) lanes 4,5 appear similar.
- The 0h and 4h ERK data for LN229 cells in Fig 4B appear similar to the 0h and 24h ERK data for LN229 cells in Fig 4C, when aspect ratio is adjusted.
- The 0h and 24h ERK data for T98G cells appear similar in Fig 4B and 4C, although the corresponding data for p-ERK and other proteins differ.
- The western blot experiments shown in Fig 4 did not include general loading controls, and the authors did not report total Rb controls for the phospho-Rb (pRb) experiments.

The authors provided pRB and ERK/p-ERK data from replication experiments and the available primary images underlying the western blots in Fig 4B, C, but the original data are no longer available for the pRB (LN229) and cyclin D1 (LN229, T98G) blots in Fig 4B; or for the cyclin D1 (U373) and ERK (LN229, T98G, U373) blots in Fig 4C. A member of our Editorial Board evaluated the data provided by authors and noted that they support the conclusions. However, owing to the lack of original primary data supporting the panels in question we have been unable to resolve the concerns raised around the pRb data in Fig 4B or the ERK data reported in Fig 4C. Furthermore, the lack of protein loading controls for the experiments in Fig 4 calls into question the validity of the results.

In light of these concerns, the *PLOS ONE* Editors retract this article.

In our assessment of this case, it also came to light that two cell lines used in the study (U87MG, U373) may be misidentified (2–4).

SFdM and PV did not agree with the retraction. GR, ETM, and JR did not respond.
