# Correction to Retraction of: EGFR Inhibition in Glioma Cells Modulates Rho Signaling to Inhibit Cell Motility and Invasion and Cooperates with Temozolomide to Reduce Cell Growth

**DOI:** 10.1371/journal.pone.0230016

**Published:** 2020-02-27

**Authors:** The PLOS ONE Editors

The retraction notice [1] for this article [2] included the following statements: ‘The western blot experiments shown in Fig 4 did not include general loading controls, and the authors did not report total Rb controls for the phospho-Rb (pRb) experiments’, ‘Furthermore, the lack of protein loading controls for the experiments in Figure 4 call into question the validity of the results.’

After publication of the notice, it was clarified that in Figure 4 of [2] “pRb” referred to total retinoblastoma protein rather than a phosphorylated form of this protein. As such, this point in the retraction notice is incorrect: “the authors did not report total Rb controls for the phospho-Rb (pRb) experiments.”

In addition, the authors noted that the total ERK and total Akt panels in Figure 4 provided information about protein loading across lanes. Additional loading controls were not requested during the re-evaluation of the article, the authors noted that such controls could in principle have been provided had they been requested.
